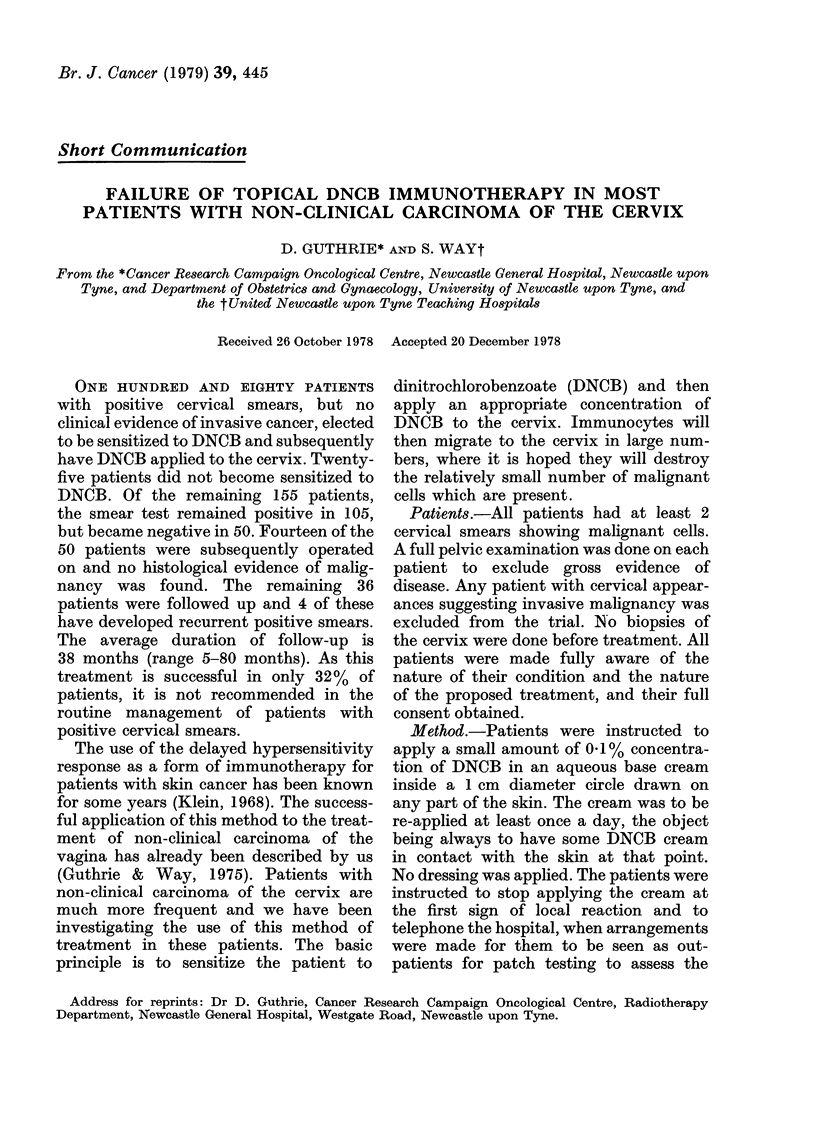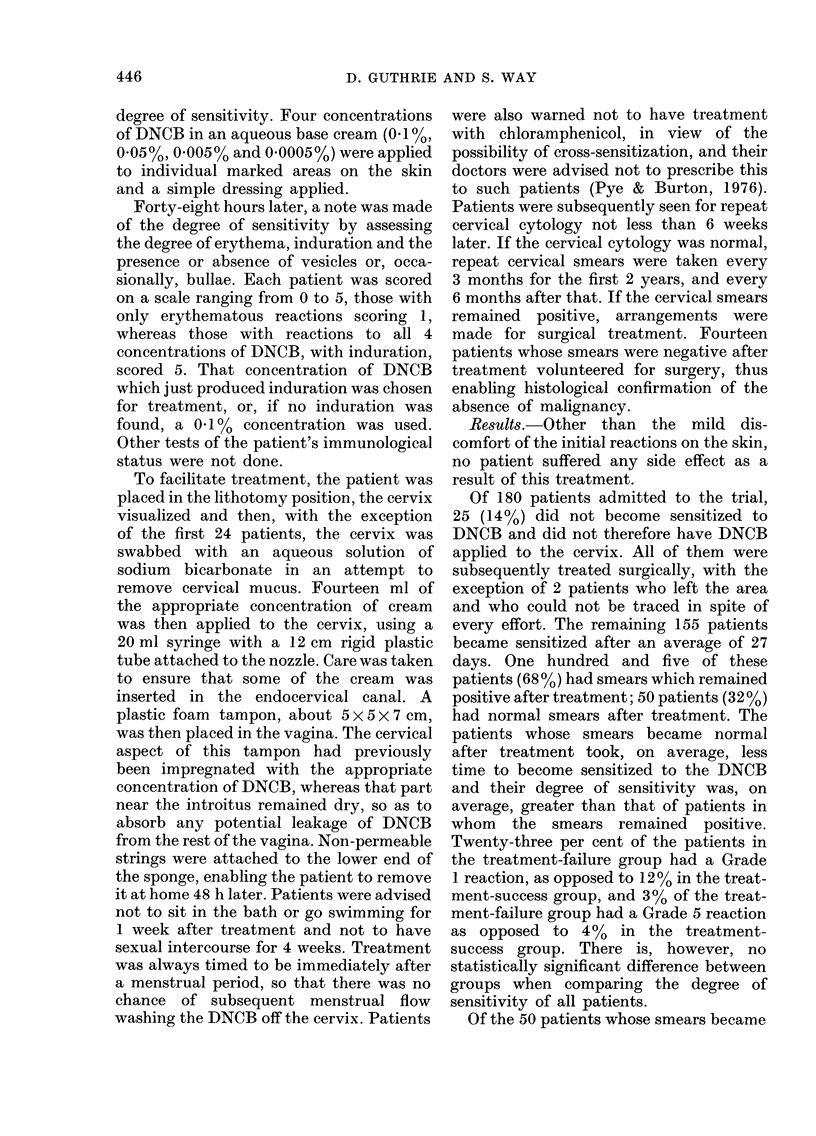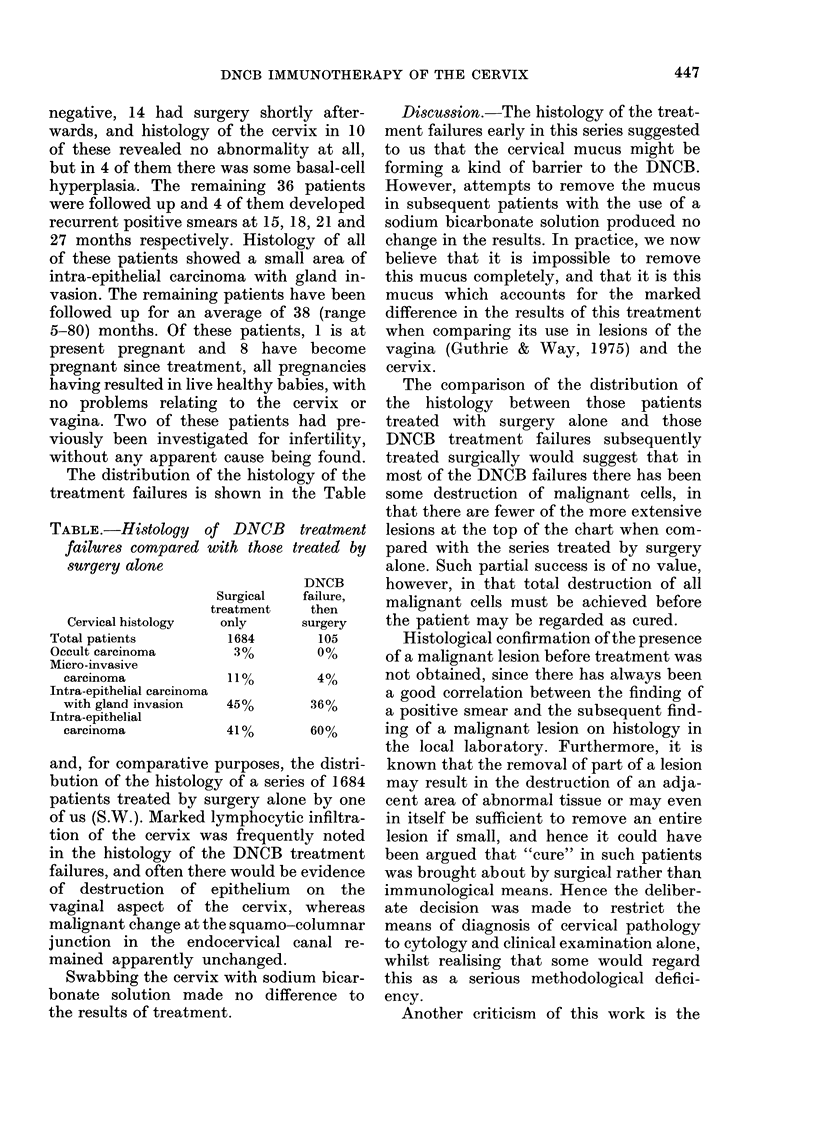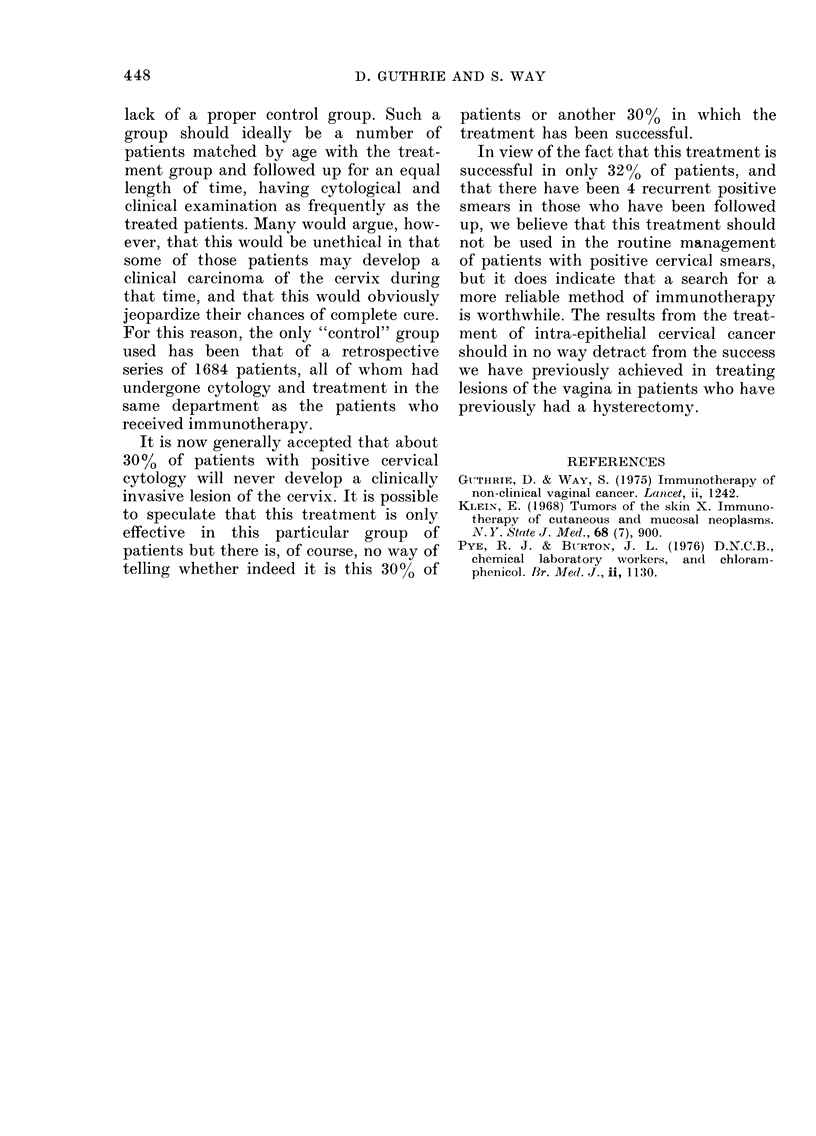# Failure of topical DNCB immunotherapy in most patients with non-clinical carcinoma of the cervix.

**DOI:** 10.1038/bjc.1979.79

**Published:** 1979-04

**Authors:** D. Guthrie, S. Way


*Br. J. Cancer* (1979) **39**, 445

***Short Communication***

### FAILURE OF TOPICAL DNCB IMMUNOTHERAPY IN MOST PATIENTS WITH NON-CLINICAL CARCINOMA OF THE CERVIX

D. GUTHRIE* AND S. WAY†

*From the *Cancer Research Campaign Oncological Centre, Newcastle General Hospital, Newcastle upon Tyne, and Department of Obstetrics and Gynaecology, University of Newcastle upon Tyne, and the †United Newcastle upon Tyne Teaching Hospitals*

Received 26 October 1978 Accepted 20 December 1978

One hundred and eighty patients with positive cervical smears, but no clinical evidence of invasive cancer, elected to be sensitized to DNCB and subsequently have DNCB applied to the cervix. Twenty-five patients did not become sensitized to DNCB. Of the remaining 155 patients, the smear test remained positive in 105, but became negative in 50. Fourteen of the 50 patients were subsequently operated on and no histological evidence of malignancy was found. The remaining 36 patients were followed up and 4 of these have developed recurrent positive smears. The average duration of follow-up is 38 months (range 5–80 months). As this treatment is successful in only 32% of patients, it is not recommended in the routine management of patients with positive cervical smears.

The use of the delayed hypersensitivity response as a form of immunotherapy for patients with skin cancer has been known for some years (Klein, 1968). The successful application of this method to the treatment of non-clinical carcinoma of the vagina has already been described by us (Guthrie & Way, 1975). Patients with non-clinical carcinoma of the cervix are much more frequent and we have been investigating the use of this method of treatment in these patients. The basic principle is to sensitize the patient to dinitrochlorobenzoate (DNCB) and then apply an appropriate concentration of DNCB to the cervix. Immunocytes will then migrate to the cervix in large numbers, where it is hoped they will destroy the relatively small number of malignant cells which are present.

*Patients.*—All patients had at least 2 cervical smears showing malignant cells. A full pelvic examination was done on each patient to exclude gross evidence of disease. Any patient with cervical appearances suggesting invasive malignancy was excluded from the trial. No biopsies of the cervix were done before treatment. All patients were made fully aware of the nature of their condition and the nature of the proposed treatment, and their full consent obtained.

*Method.*—Patients were instructed to apply a small amount of 0·1% concentration of DNCB in an aqueous base cream inside a 1 cm diameter circle drawn on any part of the skin. The cream was to be re-applied at least once a day, the object being always to have some DNCB cream in contact with the skin at that point. No dressing was applied. The patients were instructed to stop applying the cream at the first sign of local reaction and to telephone the hospital, when arrangements were made for them to be seen as out-patients for patch testing to assess the

Address for reprints: Dr D. Guthrie, Cancer Research Campaign Oncological Centre, Radiotherapy Department, Newcastle General Hospital, Westgate Road, Newcastle upon Tyne.

degree of sensitivity. Four concentrations of DNCB in an aqueous base cream (0·1%, 0·05%, 0·005% and 0·0005%) were applied to individual marked areas on the skin and a simple dressing applied.

Forty-eight hours later, a note was made of the degree of sensitivity by assessing the degree of erythema, induration and the presence or absence of vesicles or, occasionally, bullae. Each patient was scored on a scale ranging from 0 to 5, those with only erythematous reactions scoring 1, whereas those with reactions to all 4 concentrations of DNCB, with induration, scored 5. That concentration of DNCB which just produced induration was chosen for treatment, or, if no induration was found, a 0·1% concentration was used. Other tests of the patient's immunological status were not done.

To facilitate treatment, the patient was placed in the lithotomy position, the cervix visualized and then, with the exception of the first 24 patients, the cervix was swabbed with an aqueous solution of sodium bicarbonate in an attempt to remove cervical mucus. Fourteen ml of the appropriate concentration of cream was then applied to the cervix, using a 20 ml syringe with a 12 cm rigid plastic tube attached to the nozzle. Care was taken to ensure that some of the cream was inserted in the endocervical canal. A plastic foam tampon, about 5×5×7 cm, was then placed in the vagina. The cervical aspect of this tampon had previously been impregnated with the appropriate concentration of DNCB, whereas that part near the introitus remained dry, so as to absorb any potential leakage of DNCB from the rest of the vagina. Non-permeable strings were attached to the lower end of the sponge, enabling the patient to remove it at home 48 h later. Patients were advised not to sit in the bath or go swimming for 1 week after treatment and not to have sexual intercourse for 4 weeks. Treatment was always timed to be immediately after a menstrual period, so that there was no chance of subsequent menstrual flow washing the DNCB off the cervix. Patients were also warned not to have treatment with chloramphenicol, in view of the possibility of cross-sensitization, and their doctors were advised not to prescribe this to such patients (Pye & Burton, 1976). Patients were subsequently seen for repeat cervical cytology not less than 6 weeks later. If the cervical cytology was normal, repeat cervical smears were taken every 3 months for the first 2 years, and every 6 months after that. If the cervical smears remained positive, arrangements were made for surgical treatment. Fourteen patients whose smears were negative after treatment volunteered for surgery, thus enabling histological confirmation of the absence of malignancy.

*Results.*—Other than the mild discomfort of the initial reactions on the skin, no patient suffered any side effect as a result of this treatment.

Of 180 patients admitted to the trial, 25 (14%) did not become sensitized to DNCB and did not therefore have DNCB applied to the cervix. All of them were subsequently treated surgically, with the exception of 2 patients who left the area and who could not be traced in spite of every effort. The remaining 155 patients became sensitized after an average of 27 days. One hundred and five of these patients (68%) had smears which remained positive after treatment; 50 patients (32%) had normal smears after treatment. The patients whose smears became normal after treatment took, on average, less time to become sensitized to the DNCB and their degree of sensitivity was, on average, greater than that of patients in whom the smears remained positive. Twenty-three per cent of the patients in the treatment-failure group had a Grade 1 reaction, as opposed to 12% in the treatment-success group, and 3% of the treatment-failure group had a Grade 5 reaction as opposed to 4% in the treatment-success group. There is, however, no statistically significant difference between groups when comparing the degree of sensitivity of all patients.

Of the 50 patients whose smears became negative, 14 had surgery shortly afterwards, and histology of the cervix in 10 of these revealed no abnormality at all, but in 4 of them there was some basal-cell hyperplasia. The remaining 36 patients were followed up and 4 of them developed recurrent positive smears at 15, 18, 21 and 27 months respectively. Histology of all of these patients showed a small area of intra-epithelial carcinoma with gland invasion. The remaining patients have been followed up for an average of 38 (range 5–80) months. Of these patients, 1 is at present pregnant and 8 have become pregnant since treatment, all pregnancies having resulted in live healthy babies, with no problems relating to the cervix or vagina. Two of these patients had previously been investigated for infertility, without any apparent cause being found.

The distribution of the histology of the treatment failures is shown in the Table and, for comparative purposes, the distribution of the histology of a series of 1684 patients treated by surgery alone by one of us (S.W.). Marked lymphocytic infiltration of the cervix was frequently noted in the histology of the DNCB treatment failures, and often there would be evidence of destruction of epithelium on the vaginal aspect of the cervix, whereas malignant change at the squamo–columnar junction in the endocervical canal remained apparently unchanged.

TABLE.—*Histology of DNCB treatment failures compared with those treated by surgery alone*

| Cervical histology | Surgical treatment only | DNCB failure, then surgery |
|---|---|---|
| Total patients | 1684 | 105 |
| Occult carcinoma | 3% | 0% |
| Micro-invasive carcinoma | 11% | 4% |
| Intra-epithelial carcinoma with gland invasion | 45% | 36% |
| Intra-epithelial carcinoma | 41% | 60% |

Swabbing the cervix with sodium bicarbonate solution made no difference to the results of treatment.

*Discussion.*—The histology of the treatment failures early in this series suggested to us that the cervical mucus might be forming a kind of barrier to the DNCB. However, attempts to remove the mucus in subsequent patients with the use of a sodium bicarbonate solution produced no change in the results. In practice, we now believe that it is impossible to remove this mucus completely, and that it is this mucus which accounts for the marked difference in the results of this treatment when comparing its use in lesions of the vagina (Guthrie & Way, 1975) and the cervix.

The comparison of the distribution of the histology between those patients treated with surgery alone and those DNCB treatment failures subsequently treated surgically would suggest that in most of the DNCB failures there has been some destruction of malignant cells, in that there are fewer of the more extensive lesions at the top of the chart when compared with the series treated by surgery alone. Such partial success is of no value, however, in that total destruction of all malignant cells must be achieved before the patient may be regarded as cured.

Histological confirmation of the presence of a malignant lesion before treatment was not obtained, since there has always been a good correlation between the finding of a positive smear and the subsequent finding of a malignant lesion on histology in the local laboratory. Furthermore, it is known that the removal of part of a lesion may result in the destruction of an adjacent area of abnormal tissue or may even in itself be sufficient to remove an entire lesion if small, and hence it could have been argued that "cure" in such patients was brought about by surgical rather than immunological means. Hence the deliberate decision was made to restrict the means of diagnosis of cervical pathology to cytology and clinical examination alone, whilst realising that some would regard this as a serious methodological deficiency.

Another criticism of this work is the lack of a proper control group. Such a group should ideally be a number of patients matched by age with the treatment group and followed up for an equal length of time, having cytological and clinical examination as frequently as the treated patients. Many would argue, however, that this would be unethical in that some of those patients may develop a clinical carcinoma of the cervix during that time, and that this would obviously jeopardize their chances of complete cure. For this reason, the only "control" group used has been that of a retrospective series of 1684 patients, all of whom had undergone cytology and treatment in the same department as the patients who received immunotherapy.

It is now generally accepted that about 30% of patients with positive cervical cytology will never develop a clinically invasive lesion of the cervix. It is possible to speculate that this treatment is only effective in this particular group of patients but there is, of course, no way of telling whether indeed it is this 30% of patients or another 30% in which the treatment has been successful.

In view of the fact that this treatment is successful in only 32% of patients, and that there have been 4 recurrent positive smears in those who have been followed up, we believe that this treatment should not be used in the routine management of patients with positive cervical smears, but it does indicate that a search for a more reliable method of immunotherapy is worthwhile. The results from the treatment of intra-epithelial cervical cancer should in no way detract from the success we have previously achieved in treating lesions of the vagina in patients who have previously had a hysterectomy.

### REFERENCES

Guthrie, D. & Way, S. (1975) Immunotherapy of non-clinical vaginal cancer. *Lancet*, ii, 1242.

Klein, E. (1968) Tumors of the skin X. Immunotherapy of cutaneous and mucosal neoplasms. *N.Y. State J. Med.*, **68** (7), 900.

Pye, R. J. & Burton, J. L. (1976) D.N.C.B., chemical laboratory workers, and chloramphenicol. *Br. Med. J.*, **ii**, 1130.